# Tuning the Drug Release from Antibacterial Polycaprolactone/Rifampicin-Based Core–Shell Electrospun Membranes: A Proof of Concept

**DOI:** 10.1021/acsami.2c04849

**Published:** 2022-06-07

**Authors:** Martina Gruppuso, Benedetta Guagnini, Luigi Musciacchio, Francesca Bellemo, Gianluca Turco, Davide Porrelli

**Affiliations:** † Department of Medicine, Surgery and Health Sciences, University of Trieste, Piazza dell’Ospitale 1, 34129 Trieste, Italy; ‡ Department of Engineering and Architecture, University of Trieste, Via Alfonso Valerio 6/1, 34127 Trieste, Italy

**Keywords:** antibacterial, coaxial, drug release, electrospinning, polycaprolactone, rifampicin

## Abstract

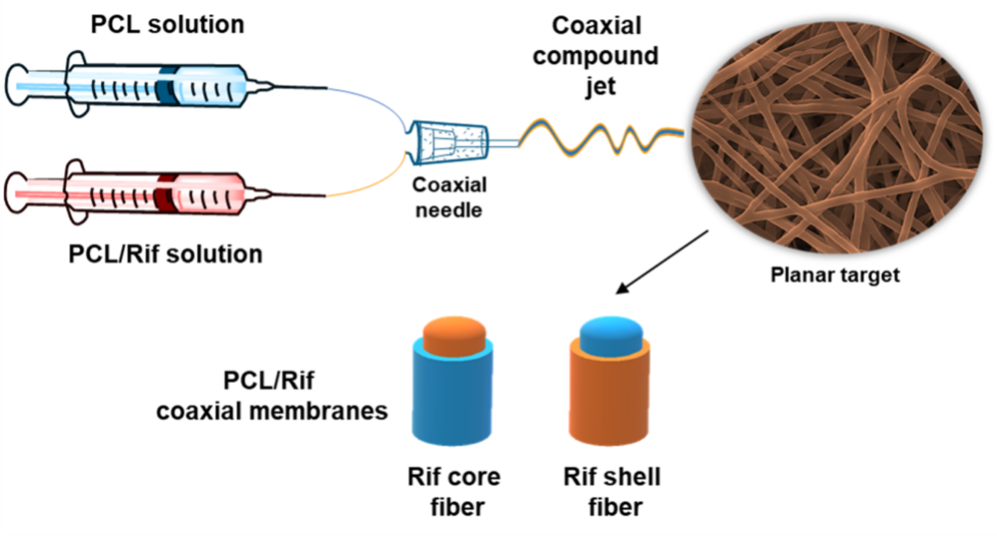

The employment of coaxial fibers for guided tissue regeneration can be extremely advantageous since they allow the functionalization with bioactive compounds to be preserved and released with a long-term efficacy. Antibacterial coaxial membranes based on poly-ε-caprolactone (PCL) and rifampicin (Rif) were synthesized here, by analyzing the effects of loading the drug within the core or on the shell layer with respect to non-coaxial matrices. The membranes were, therefore, characterized for their surface properties in addition to analyzing drug release, antibacterial efficacy, and biocompatibility. The results showed that the lower drug surface density in coaxial fibers hinders the interaction with serum proteins, resulting in a hydrophobic behavior compared to non-coaxial mats. The air-plasma treatment increased their hydrophilicity, although it induced rifampicin degradation. Moreover, the substantially lower release of coaxial fibers influenced the antibacterial efficacy, tested against *Escherichia coli*, *Staphylococcus aureus*, and *Pseudomonas aeruginosa*. Indeed, the coaxial matrices were inhibitory and bactericidal only against *S. aureus*, while the higher release from non-coaxial mats rendered them active even against *E. coli*. The biocompatibility of the released rifampicin was assessed too on murine fibroblasts, revealing no cytotoxic effects. Hence, the presented coaxial system should be further optimized to tune the drug release according to the antibacterial effectiveness.

## 1 Introduction

Regenerative medicine aims at restoring, maintaining, or improving tissue function and esthetics, availing substitutes that interact with the surrounding environment and actively stimulate tissue regeneration without replacing it.^1,2^ This goal can be achieved by producing biomaterials able to mimic the structure of the tissue to be restored avoiding any adverse response, thus promoting the so-called guided tissue regeneration (GTR).^3−7^

In this context, the electrospinning technique can be particularly useful, since it allows the production of micro-nanostructured membranes with interconnected fibers recalling the extracellular matrix (ECM) architecture.^8−10^ The simplest set-up consists of the production of nanofibers by extruding a polymeric solution under an electric field.^11−14^ This allows obtaining three-dimensional (3D) nanofibrous mats, with different characteristics depending on the polymers chosen, as well as on process and environmental parameters (*i.e.*, the applied voltage, the flow rate, the distance between the needle and the collector, the temperature, or the humidity).^15−17^ The nanofibrous meshes can be easily functionalized with bioactive cues, whose release can be regulated through the specific surface area, porosity, and fiber diameter, further promoting the structural and functional recovery of the injured site.^18−20^

To favor the inclusion and to preserve and tune at the same time the release of the loaded drugs or bioactive molecules, the electrospinning set-up can be implemented using coaxial needles,^21−24^ which allow to produce fibers characterized by a radial and concentric alignment along the transversal section of the fiber, thus defined “core–shell”.^25,26^ The miscibility of the polymers employed is of pivotal importance since in the presence of immiscible or semi-miscible solutions a more stable Taylor cone is created; moreover, the compatibility of the solutions, the polymer ratio, and the differential flow rate between the core and shell are relevant parameters in the production of well-defined coaxial fibers.^27−29^ The core–shell structure can enable a sustained and/or two-stage release, avoiding the burst and rapid release of the drugs. Moreover, multiple drugs and bioactive moieties can be differentially incorporated into the core or shell layer, depending on the stability of the selected compound and on the desired release kinetics.^13,30−32^

Synthetic polymers (such as polyurethane, poly(lactic acid), poly(vinyl alcohol), and poly-ε-caprolactone) have been widely exploited during the last 15 years and their employment has been approved by the FDA for clinical purposes. Indeed, they allow the production of biocompatible medical devices with excellent properties to guide tissue regeneration, such as mechanical strength, thermal stability, or a good degradation profile.^33−35^ However, they are basically hydrophobic and lack any intrinsic bioactivity thus requiring surface modifications and/or the addition of bioactive.^36−38^ Among the most used synthetic polymers, poly-ε-caprolactone (PCL) stands out as a low-cost, biocompatible polymer with suitable properties for long-term implantation. Indeed, it is a semicrystalline linear aliphatic polyester, which is slowly degraded (2–4 years) by hydrolysis of the ester linkages under physiological conditions, while maintaining high mechanical properties.^39−41^ A wide variety of PCL-based electrospun scaffolds has been produced for different biomedical purposes, such as guided bone regeneration, neuronal tissue engineering, tendon regeneration, anticancer therapy, or wound management.^42−47^ Thanks to its versatility and ease of processability, PCL has also been employed for the production of coaxial matrices, both in combination with synthetic or natural polymers, providing satisfactory results.^48−54^

When considering biomaterials for tissue regeneration, the risk of infections represents the most serious complication hindering the structural and functional recovery of the disrupted tissue.^55−57^ For this reason, functionalization with antibacterial compounds, such as antibiotics or antimicrobial peptides, could be crucial in guaranteeing the restoration of the injured site.^58−61^ Among them, rifampicin is one of the most effective broad-spectrum bactericidal antibiotics, which blocks the RNA-polymerase β subunit, inhibiting the transcription and subsequent bacterial protein synthesis.^62,63^ It demonstrated its activity against both Gram-positive and Gram-negative bacteria, being particularly effective in the case of biofilm formation by staphylococci.^64−66^ Several polymers, such as poly(lactic acid), poly(vinyl alcohol), or poly(lactic acid-co-glycolic acid) have already been studied to produce rifampicin-based antibacterial nanofibrous structures.^67−69^ Among them, even rifampicin-loaded PCL membranes for orthopedic application have been characterized, revealing their antibacterial effect against *Pseudomonas aeruginosa* and *Staphylococcus epidermis* in the first 6 h.^70^

As a proof of concept, the present study reports the possibility of tuning drug release from core–shell electrospun fibers. PCL was designated as a building polymer, and rifampicin was chosen to confer antibacterial properties to the coaxial membranes, and was differentially loaded in the core or shell layer. The surface properties of the membranes were assessed in terms of wettability and surface free energy. The drug-release kinetics was then studied to prove the presence of the coaxial structure and demonstrate how the localization of the antibiotic influences its release. The biocompatibility of the core–shell fibers was tested too on a murine fibroblast cell line, evaluating the proliferation rate of cells in the presence of the biomaterial. The antibacterial efficacy of the rifampicin-membrane was finally examined against three of the most common bacterial strains involved in the infections of biomaterials and implants, namely *Escherichia coli*, *Staphylococcus aureus*, and *P. aeruginosa*. As a comparison, all of the tests were even carried out on PCL-rifampicin non-coaxial nanofibers, evaluating the benefits of using coaxial electrospinning with respect to the traditional set-up.

## 2 Materials and Methods

### 2.1 Materials

Polycaprolactone (PCL, *M*_w_ = 80000), dichloromethane (DCM), *N*,*N*-dimethylformamide (DMF), methanol, and chloroform were purchased from Sigma-Aldrich (St. Louis). Glass syringes (with an inner diameter of 14.6 mm), three-layers coaxial needle, and coaxial kit were acquired from Linari NanoTech (Pisa, Italy). The KDS-100-CE syringe pump was purchased from KD Scientific (Holliston, MA). Rifampicin was acquired from EMD Millipore Corp. The D-ES30PN-20W potential generator was purchased from Gamma High Voltage Research Inc. (Ormond Beach, FL). Recombinant Trypsin–EDTA 1X, penicillin/streptomycin 100X, l-glutamine 100X, fetal bovine serum (FBS), and Dulbecco’s modified Eagle’s medium (DMEM) were purchased from Euroclone (Milan, Italy).

### 2.2 Membrane Preparation

#### 2.2.1 Single-Needle Membranes

To produce single-needle membranes, 12% (w/V) PCL was solubilized in DCM:DMF (ratio 7:3), first PCL was dissolved in DCM overnight under magnetic stirring and DMF was added to the solution the day after. Electrospun PCL membranes (hereafter named “**CTRL no coax**”) were produced with the following experimental set-up: time of the process, 1 h; flow rate, 2 mL/h; voltage, 17 kV; needle diameter, 21 G; needle-collector distance, 15 cm; negative pole to the target. Rifampicin-loaded single-needle membranes (from this point called “**Rif no coax**”) were prepared using the same experimental set-up. In this case, 0.1% (w/V) rifampicin was solubilized in DMF, prior to the addition of DMF to the PCL in DCM solution. Considering rifampicin light sensitivity, all of the procedure was performed under dark conditions.

#### 2.2.2 Coaxial Membranes

**Figure 1:**
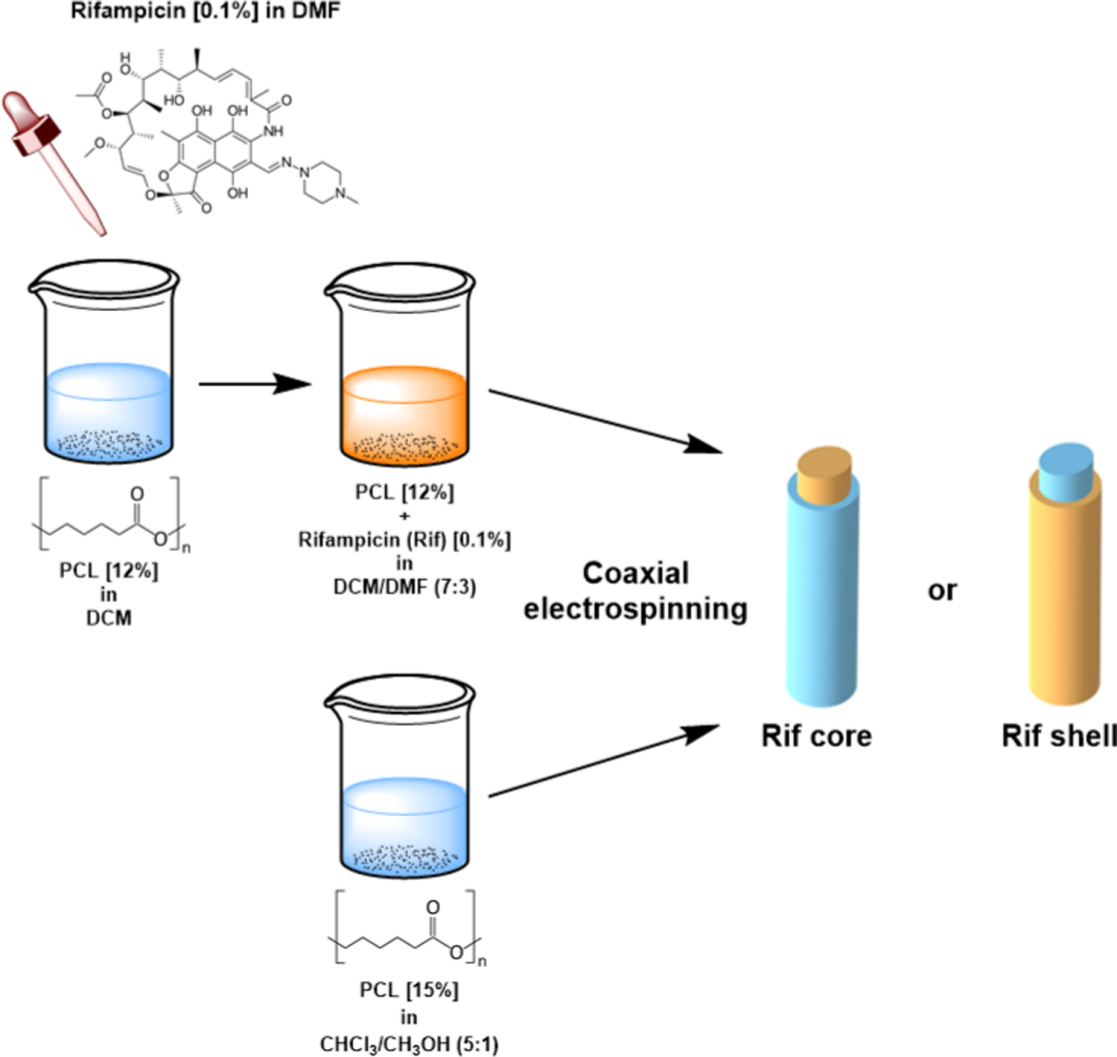
Schematic representation of the preparation method to obtain coaxial membranes with the rifampicin included in the core or incorporated in the shell of the fiber.

Coaxial membranes were produced by alternatively introducing rifampicin in the core or shell layer, hereafter indicated as “**Rif core**” and “**Rif shell**”, respectively. The rifampicin-containing solution was the same as described in Section 2.2.1, namely, 12% (w/V) PCL in DCM:DMF (7:3) with 0.1% (w/V) rifampicin. The second solution used for coaxial membrane production was obtained by first dissolving 15% (w/V) PCL in chloroform under magnetic stirring and adding methanol to the solution the day after. The respective control membranes are represented by coaxial fibers without rifampicin, where the 12% (w/V) PCL solution was placed in the core or shell layer (from now on named “**CTRL core**” and “**CTRL shell**”, respectively). Electrospun **Rif core** membranes were obtained under dark conditions through the following experimental set-up: time of the process, 10 min; core solution flow rate, 1 mL/h; shell solution flow rate; 3 mL/h; voltage, 30 kV; inner needle diameter, 21 G; outer needle diameter, 15 G; needle-collector distance, 21 cm; negative pole to the target. Electrospun **Rif shell** membranes were produced under dark conditions too, with a different experimental set-up: time of the process, 10 min; core solution flow rate, 3.5 mL/h; shell solution flow rate, 2 mL/h; voltage, 30 kV; inner needle diameter, 21 G; outer needle diameter, 15 G; distance needle-collector, 24 cm; negative pole to the target. The control membranes were produced with the same protocols as those used for their rifampicin-loaded counterpart. A schematic representation of the preparation protocol is reported in Figure 1.

### 2.3 Air-Plasma Treatment

Some of the single-needle and coaxial membranes were subjected to air-plasma treatment to improve their hydrophilicity. The process was performed using a PDC-32G plasma cleaner (Harrick Plasma, Ithaca NY) used in low power (6.8 W) with a pressure of 0.1 mTorr for 5 min, by adapting the protocol presented by Can-Herrera and co-workers.^71^

### 2.4 Scanning Electron Microscopy (SEM)

To analyze fiber morphology, membrane samples were placed on aluminum stubs covered with a double-sided carbon tape and sputter-coated with gold using a Sputter Coater K550X (Emitech, Quorum Technologies Ltd, U.K.). They were then analyzed using a scanning electron microscope (Quanta 250 SEM, FEI, Oregon), working in secondary electron detection mode. The acceleration voltage was set at 25–30 kV, while the working distance was set at 10 mm to obtain optimal magnification. Fiber diameters were calculated using Fiji software,^72^ by randomly selecting 50 fibers from each sample.

### 2.5 Contact Angle and Surface Free Energy Analyses

The wettability of membrane samples was assessed by measuring the contact angle, using the sessile drop method. All types of membranes were tested, by analyzing 6 samples for each condition. The contact angle was measured on images acquired with an optical microscope (Leica MZ16) equipped with a 45° tilted mirror and a digital camera (Leica DFC 320), then connected to the software Image Pro 3D Suite. Membrane behavior was examined in the presence of three types of fluids, namely deionized water (DW), deionized water + 10% FBS, and DMEM. For each type of fluid, 4 μL were deposited on the sample and the images were obtained after 30 s to allow drop stabilization. The acquired images were analyzed using Fiji software and the contact angle of each type of sample in the presence of all types of fluid was calculated. Surface free energies were evaluated using the Owens–Wendt method^73^ adapted by Ren et al.^74^ and Can-Herrera et al.^71^ Both DW and ethylene glycol (EG) contact angles (4 μL of fluid/sample) were considered to calculate the surface energy components: the polar/hydrophilic component (γ_s_^p^) and the dispersive/hydrophobic component (γ_s_^d^). The total surface free energy (γ_s_) was, therefore, calculated as
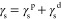
1

### 2.6 *In Vitro* Release of Rifampicin

The release of rifampicin from air-plasma-treated and not treated membranes (diameter, 8 mm) was studied UV spectrophotometry (Ultraspec 2100 pro, Amersham Bioscience) at 237 nm. To evaluate the release of rifampicin, 3 samples for each condition were tested and placed in 24-well plates, by adding 500 μL of saline phosphate buffer (PBS) as the release medium. After 1, 4, 24, 48, and 72 h, 500 μL of solution were collected from each well for absorbance evaluation and the release solution was substituted with fresh PBS at each time point. The plates were incubated at 37 °C under dark conditions, maintaining a wet environment to avoid PBS evaporation. The released rifampicin was quantified using the Lambert–Beer equation, knowing that ε_237nm_ is equal to 33 200, and normalized on 1 mg of the membrane.

### 2.7 Cell Culture

Murine fibroblasts (NIH/3T3, ATCC CRL-1658) were cultured in high-glucose DMEM supplemented with 10% FBS, 2 mM l-glutamine, 100 U/mL penicillin, and 0.1 mg/mL streptomycin under a humid atmosphere at 37 °C and with 5% pCO_2_. Cells were passed, using 0.25% trypsin, three times a week or when the confluence level was estimated at about 70–80% of the available culture space.

#### 2.7.1 Proliferation Assay

All types of membranes were cut in disks (diameters, 8 mm) and sterilized with UV irradiation for (i) 10 min in the case of rifampicin-loaded mats and (ii) 45 min in the case of the controls without rifampicin. Cells at a concentration of 10 000 cells/well, suspended in 1 mL of complete high-glucose DMEM, were seeded onto 24-well cell culture plates and then incubated at 37 °C with 5% pCO_2_. After 4 h of incubation, both treated and not treated membrane samples were added to cell-containing wells, to evaluate cell behavior in the presence of the material. Cells seeded in the absence of material were tested as proliferation control, while empty wells with the culture medium only were used as blank. Cell proliferation was evaluated after 1, 4, and 7 days using a Resazurin Cell Viability Assay Kit (Sigma-Aldrich, St. Louis), by testing five samples for each type of membrane. At each time point, the medium was removed and 400 μL of Resazurin solution (diluted 1/30 in culture medium) was added to the wells. After 4 h of incubation, 200 μL of the Resazurin solution was collected from each well and transferred to a black plate for fluorescence reading. After that, each well was washed with PBS and replaced with 1 mL of fresh medium. The fluorescence was analyzed using a spectrofluorometer GloMax Multi+ Detection System (Promega, Madison, WI) with an excitation wavelength of 525 nm and an emission wavelength in the range 580–640 nm.

### 2.8 Antibacterial Assays

#### 2.8.1 Bacterial Strains

*E. coli* (ATCC 25923), *S. aureus* (ATCC 25922), and *P. aeruginosa* (ATCC 27853) were swiped on Mueller–Hinton (MH) agar plates (MHA; Oxoid S.p.A., Milan, Italy) from a glycerol stock at −80 °C and grown overnight at 37 °C. For liquid culture, some bacterial colonies were thereafter collected from the Petri plates and resuspended in 4 mL of MH medium. Each bacterial inoculum was then incubated overnight at 37 °C under agitation (140 rpm). The day after, a re-inoculum was prepared by diluting an aliquot of overnight cultures (300 μL of each bacterial strain) in 10 mL of fresh MH medium and then incubating it at 37 °C and 140 rpm for about 90 min (up to mid-log phase), until an optical density at 600 nm (OD_600_) of approximately 0.3 was achieved. The bacterial concentration was, therefore, evaluated based on predictive models, knowing that (i) OD_600_ = 0.31 indicates a bacterial concentration of 4.6 × 10^7^ CFU/mL for *E. coli*, (ii) OD_600_ = 0.1 means a bacterial concentration of 5 × 10^8^ CFU/mL in the case of *S. aureus*, and (iii) OD_600_ = 0.3 implies a bacterial concentration of 1.5 × 10^7^ CFU/mL for *P. aeruginosa*.

#### 2.8.2 Minimal Inhibitory Concentration (MIC) and Minimal Bactericidal Concentration (MBS) Assays

The antibacterial efficacy was evaluated in the case of not treated membranes (diameter, 8 mm). PCL/Rif samples were placed in a 48-well culture plate under dark conditions, while PCL-based membranes were sterilized under UV irradiations for 30 min and then placed on a 48-well plate. Based on the initial concentration, bacterial strains were diluted in the MH medium to obtain a final concentration of 2.5 × 10^5^ CFU/mL and the minimal inhibitory concentration (MIC) assay was performed. One sample for each type of membrane was tested, namely **CTRL no coax**, **Rif no coax**, **CTRL core**, **Rif core**, **CTRL shell**, and **Rif shell**. In each case, 380 μL of the diluted bacterial suspension were added to the wells to cover the membranes, paying attention to the dark conditions so as to prevent degradation of rifampicin upon light exposure. On the other hand, 380 μL of MH medium and 380 μL of bacterial culture without membranes were considered as negative control and growth control, respectively. The plate was sealed with Parafilm to minimize the evaporation and incubated overnight in the dark at 37 °C. The eventual inhibitory effect of the incubated membranes was analyzed after 18 h, by first observing medium turbidity. After that, to effectively evaluate the bacterial growth in the presence of the material, 200 μL of bacterial suspension were collected from each well and diluted with 800 μL of MH medium, thereby measuring the absorbance at 600 nm. The inhibition of bacterial growth was evaluated by comparing the absorbance of the bacterial suspensions growing in the presence of membranes with the bacterial suspension without membranes.

Furthermore, to investigate the presence of any bactericidal effect, the minimal bactericidal concentration (MBC) assay was carried out on *S. aureus*. In this case, 25 μL were collected from each well and spread on Petri dishes containing the MH-agar (Sigma-Aldrich) medium. The plates were then incubated overnight at 37 °C and after 18 h the growing colonies were counted to assess the possible bactericidal effect of the tested membranes.

### 2.9 Statistical Analysis

Statistical analyses were performed using GraphPad software (Graphpad Holdings, LLC). Data satisfying normality (Shapiro–Wilk test) assumptions were analyzed by means of two-way analysis of variance (ANOVA) test, applying Bonferroni’s correction. Data that did not satisfy the normality assumption were examined by Kruskal–Wallis and Mann–Whitney non-parametric tests, applying Bonferroni’s correction. Statistical significance was pre-set at α = 0.05.

## 3 Results

### 3.1 Electrospun Fiber Morphology

Antibacterial PCL-based coaxial fibers were produced using rifampicin (Rif) as an antibacterial agent. By varying the coaxial configuration, the antibiotic was alternatively loaded into the core or shell layer, with the purpose of evaluating the differential effect of the drug based on its location; membranes thus produced will be named **Rif core** and **Rif shell**, respectively. At the same time, single-needle PCL/Rif membranes (here named **Rif no coax**) were synthesized to analyze the differences between coaxial and non-coaxial systems in terms of drug incorporation and almost immediate availability. In all cases, PCL membranes without rifampicin were manufactured as controls (here named, **CTRL core**, **CTRL shell**, **CTRL no coax**).

**Figure 2:**
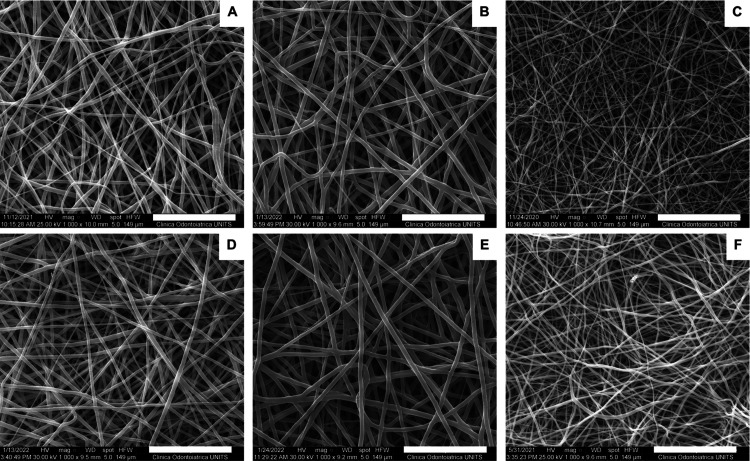
SEM micrographs of coaxial and single-needle PCL-based membranes with or without rifampicin: (A) **CTRL core**, (B) **CTRL shell**, (C) **CTRL no coax**, (D) **Rif core**, (E) **Rif shell**, and (F) **Rif no coax**. Scale bar: 50 μm.

All of the membranes revealed an optimal morphology, with homogenous, defect-free, and randomly oriented fibers (Figure 2).

**Figure 3:**
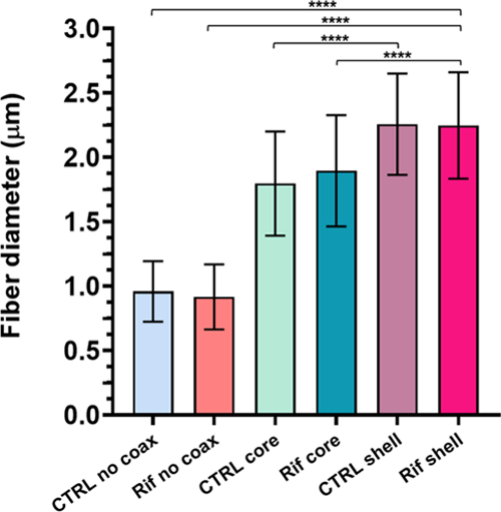
Comparison of fiber diameters between all types of membranes. Statistical analysis was performed with Kruskal–Wallis and Mann–Whitney test for a comparison of two-groups, applying Bonferroni’s correction. Statistically significant differences are indicated as asterisks (*).

The fiber diameter was calculated by randomly selecting 50 fibers on each type of sample. The results (Figure 3) showed that the mean fiber diameter of single-needle membranes is significantly lower than coaxial matrices. Moreover, the incorporation of rifampicin in the shell significantly alters the morphology, with an increase in the fiber diameter (2.25 ± 0.41 μm) with respect to rifampicin inclusion in the core (1.89 ± 0.43 μm). This is also true in the case of the respective controls, where **CTRL shell** membranes have a higher mean diameter (2.26 ± 0.39 μm) with respect to **CTRL core** (1.8 ± 0.4 μm). Such behavior is probably due to the higher flow rates used to produce **CTRL shell** and **Rif shell** membranes with respect to **CTRL core** and **Rif core**. On the other hand, the presence of rifampicin does not significantly influence fiber morphology if compared only with the PCL controls.

### 3.2 Wettability and Surface Free Energy of Electrospun Membranes

**Figure 4:**
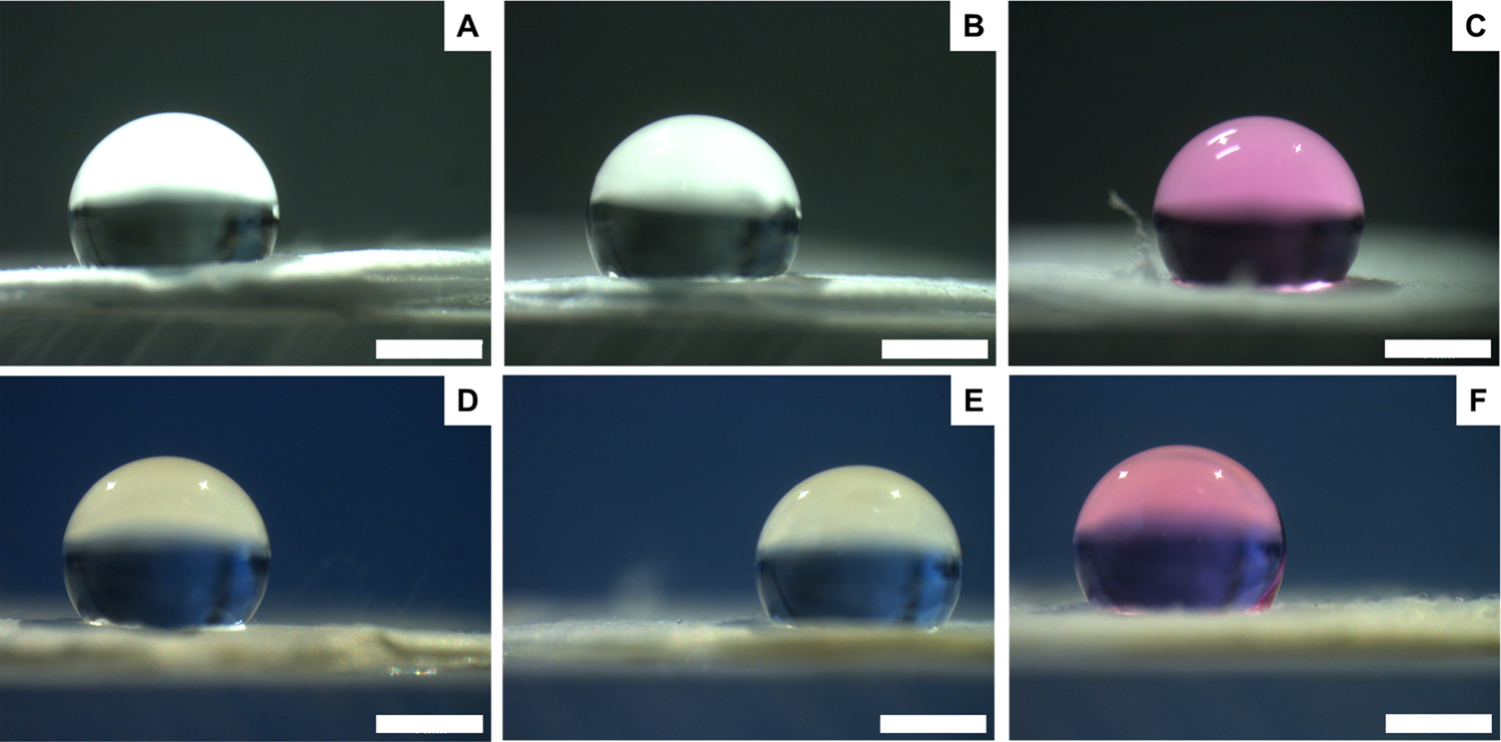
Contact angle images of **CTRL core** (A–C) and **Rif core** (D–F) membranes measured in the presence of three types of fluids: DW (left), DW + 10% FBS (center), and DMEM (right). Scale bar: 1 mm.

**Figure 5:**
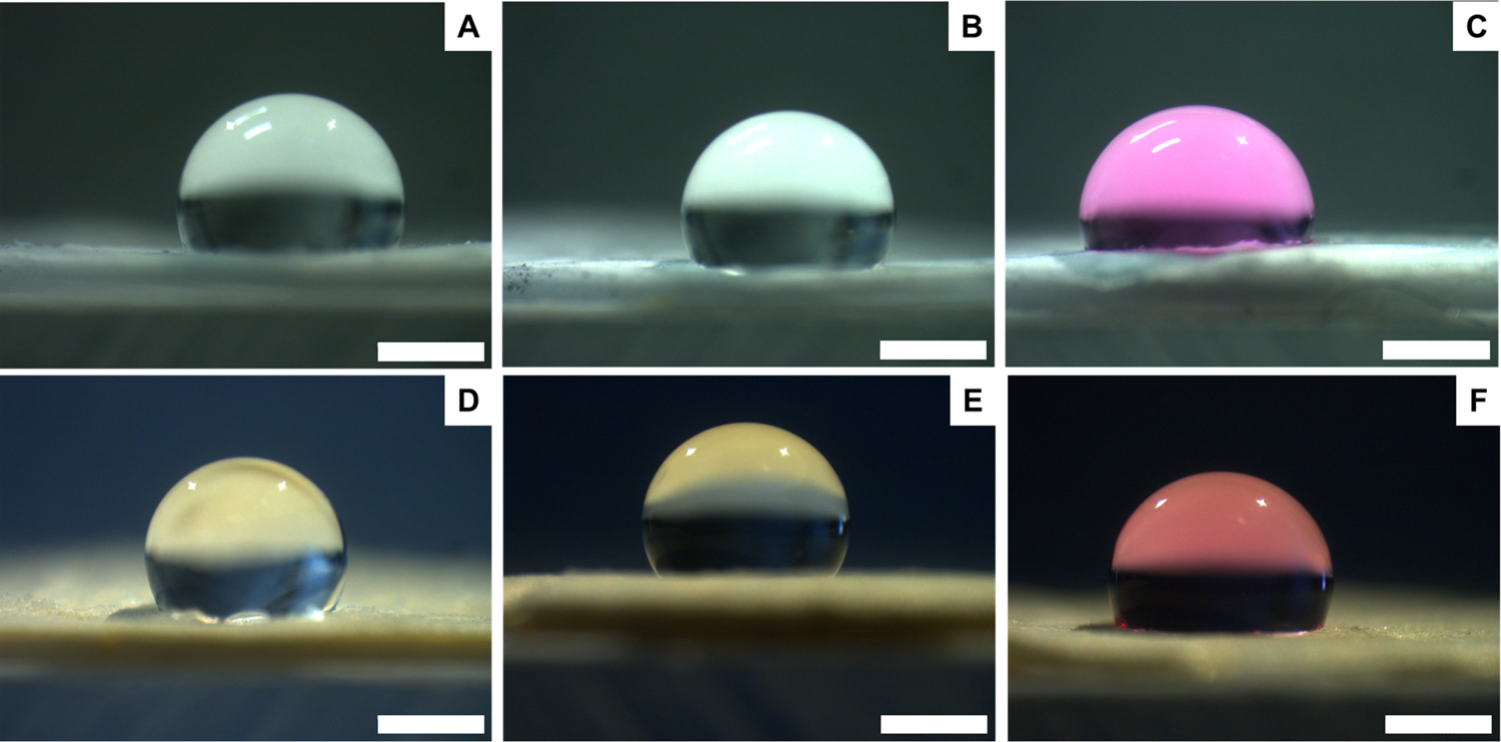
Contact angle images of **CTRL shell** (A–C) and **Rif shell** (D–F) membranes measured in the presence of three types of fluids: DW (left), DW + 10% FBS (center), and DMEM (right). Scale bar: 1 mm.

The wettability of all types of electrospun membranes, coaxial and non-coaxial, was studied by means of contact angle measurements in the presence of three types of fluids: (i) deionized water (DW), (ii) DW added with 10% fetal bovine serum (FBS), to evaluate any possible interaction with serum proteins, and (iii) Dulbecco’s modified Eagle medium (DMEM), to assess membrane behavior in an *in vitro* cellular environment. Both not treated and plasma-treated membranes were tested to examine the effectiveness of air-plasma treatment in increasing PCL-based membrane hydrophilicity. In the case of not treated coaxial membranes, both the samples with rifampicin included in the core (Figure 4) and incorporated in the shell (Figure 5) revealed a basically hydrophobic behavior in the presence of all types of fluids.

**Figure 6:**
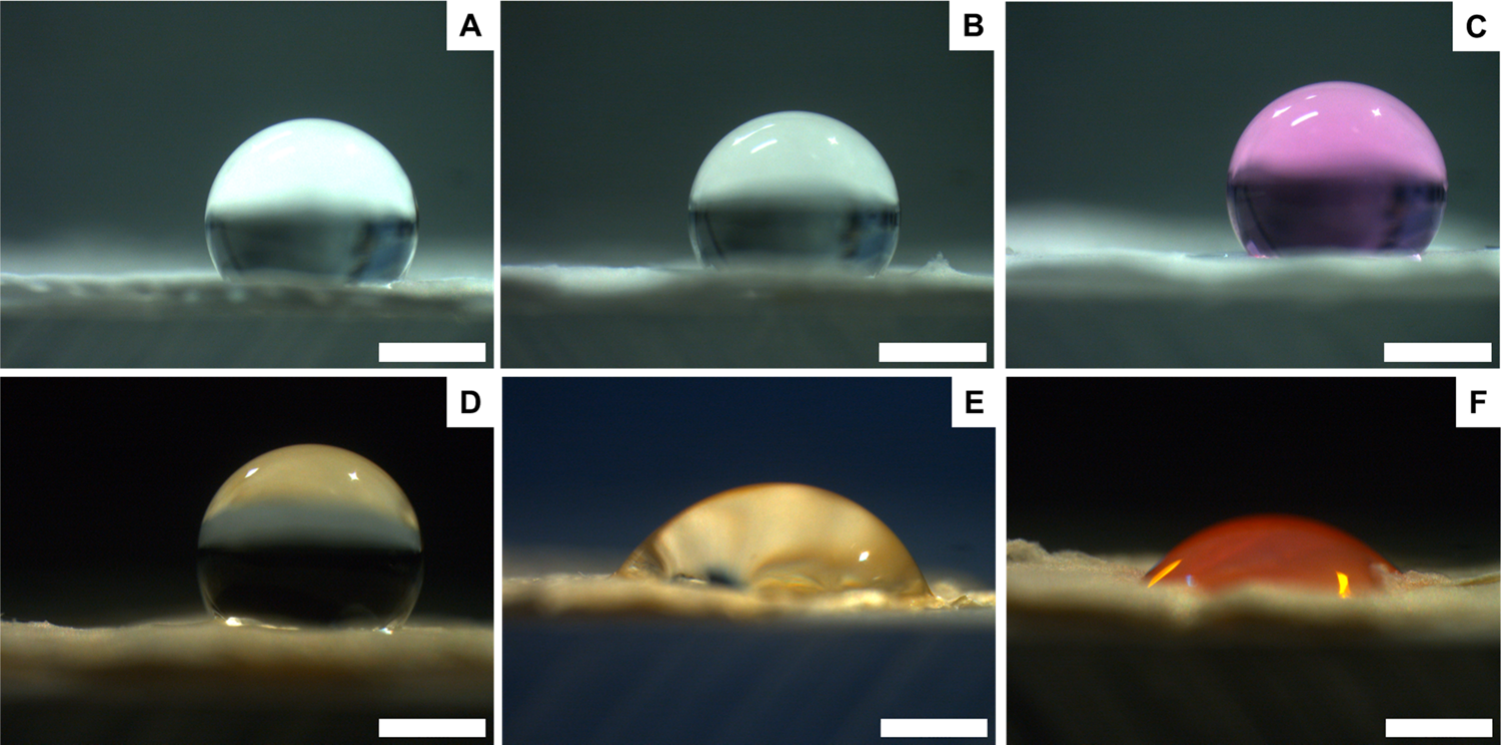
Contact angle images of **CTRL no coax** (A–C) and **Rif no coax** (D–F) membranes measured in the presence of three types of fluids: DW (left), water + 10% FBS (center), and DMEM (right). Scale bar: 1 mm.

On the other hand, not treated **Rif no coax** membranes revealed the expected hydrophobic behavior in the presence of DW (Figure 6D), but they were hydrophilic in the case of DW + FBS and DMEM (Figure 6E,F). This is probably due to the presence of rifampicin, which is highly available in the non-coaxial structure due to the lower fiber diameter, largely interacting with serum proteins and increasing electrospun mat hydrophilicity.

The same analysis was carried out on both coaxial and non-coaxial plasma-treated samples, revealing in all cases a total wettability for all types of fluids tested (Figures S1–S3), indicating the effectiveness of this type of treatment in modifying the surface properties of PCL-based membranes.

Subsequently, to evaluate the surface free energy of the samples according to the Owens–Wendt method, the contact angle of water was used to calculate the polar/hydrophilic component (γ_s_^p^), while the contact angle of ethylene glycol (EG) was examined to estimate the dispersive/hydrophobic component (γ_s_^d^). The analysis was performed both in the case of not treated and plasma-treated electrospun mats. The high contact angle values (>90°), except for **Rif no coax** in the presence of DW + FBS and DMEM, along with the low values of the polar component (γ_s_^p^) confirmed the basically hydrophobic behavior of not treated membranes (Table 1).

**Table 1:** Mean Values of the Contact Angle Measurements and Surface Free Energy Components for Not Treated PCL and PCL/Rif Membranes^a^

	contact angle DW [deg]	contact angle DW + FBS [10%] [deg]	contact angle DMEM [deg]	γ_s_^d^ [mJ/m^2^]	γ_s_^p^ [mJ/m^2^]	γ_s_ [mJ/m^2^]
**Rif core**	107.70 ± 7.02	105.13 ± 6.00	97.35 ± 15.06	79.48 ± 0.1	5.48 ± 2.77	84.96
**CTRL core**	105.52 ± 7.94	111.05 ± 11.47	100.43 ± 7.27	79.48 ± 0.1	4.78 ± 2.46	84.26
**Rif shell**	101.97 ± 10.73	107.34 ± 8.20	98.53 ± 5.84	79.48 ± 0.1	3.95 ± 2.37	83.44
**CTRL shell**	107.15 ± 0.90	103.52 ± 5.14	102.68 ± 3.27	79.48 ± 0.1	5.00 ± 0.33	84.48
**Rif no coax**	105.18 ± 10.82	61.82 ± 7.54	9.24 ± 19.48	79.48 ± 0.1	4.98 ± 3.41	84.46
**CTRL no coax**	114.50 ± 2.83	101.46 ± 14.54	115.16 ± 3.89	79.48 ± 0.1	8.13 ± 1.36	87.61

a EG contact angle values are all equal to 0°.

On the other hand, in the case of plasma-treated membranes, contact angles equal to 0° were registered in all cases, with the polar component (γ_s_^p^ = 19.06 ± 0.1 mJ/m^2^) being higher than not treated membranes, according to the increase in membrane hydrophilicity. The dispersive/hydrophobic component (γ_s_^d^) resulted to be equal for all of the tested membranes (79.48 ± 0.1 mJ/m^2^), as well as the total surface free energy (γ_s_ = 98.54 mJ/m^2^).

### 3.3 Rifampicin Release Kinetics

The release of rifampicin from PCL/Rif membranes was evaluated to estimate how the differential drug loading together with the employment of distinct structures (namely, coaxial and non-coaxial) can influence its release in time. In addition, plasma-treated matrices were compared with not treated ones to observe the effects of the activation process on the integrity and effectiveness of the antibiotic incorporated.

**Figure 7:**
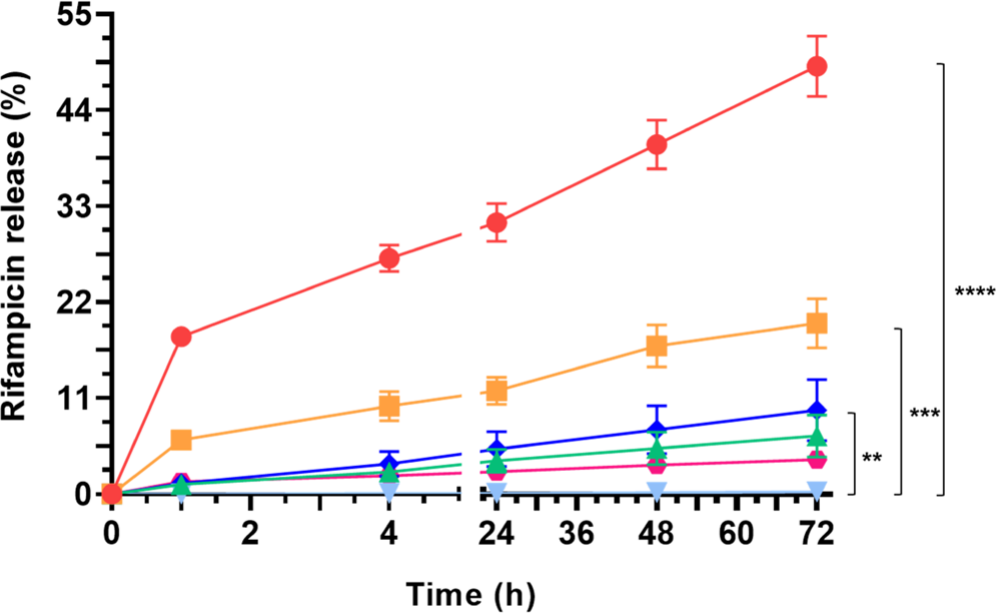
Rifampicin release kinetic from not treated **Rif no coax** (solid red circle), **Rif core** (solid green triangle up), **Rif shell** (solid blue diamond), plasma-treated **Rif no coax** (solid orange box), **Rif core** (light blue triangle down), and **Rif shell** (solid magenta hexagon). The released rifampicin was quantified using the Lambert–Beer equation, knowing that ε_237nm_ is equal to 33 200, and normalized on 1 mg of membrane. Statistical analysis was performed through two-way ANOVA, applying Bonferroni’s correction. Statistically significant differences are indicated as asterisks (*).

As shown in Figure 7, the amount of antibiotic released from not treated **Rif no coax** is significantly higher (49% after 72 h) than the other types of membranes tested. The higher release with respect to plasma-treated **Rif no coax** (20% after 72 h) is due to a lower amount of intact rifampicin within the nanofibrous mesh after the activation process. Indeed, this type of treatment seems to partially degrade the drug, leading to a significantly lower release in time. On the other hand, the difference between the non-coaxial and coaxial structures could be first explained by the lower diameter of non-coaxial nanofibers since it leads to an increase in the surface density of rifampicin available and immediately released from the biomaterial. Second, the inclusion of rifampicin in the core layer protects it from the external environment, allowing a slower and prolonged release in time with respect to non-coaxial matrices or membranes with the drug incorporated in the shell layer. Therefore, the assumption of fiber coaxial structure is proved by the differential antibiotic release from the core (6.6% after 72 h for not treated membranes) or shell (9.6% after 72 h for not treated membranes) layers, with the shell layer releasing slightly faster. It should be noted that the inclusion of rifampicin in the core of the fibers does not protect it from the air-plasma degradation, leading to an almost absent release within the first 72 h (0.3%), affecting its possible antibacterial action.

### 3.4 Electrospun Mat Biocompatibility

The biocompatibility of PCL/Rif membranes, both non-activated or plasma-treated, was assessed by evaluating NIH/3T3 proliferation in the presence of the biomaterial after 1, 4, and 7 days of exposition, with the aim to verify the possible cytotoxic effects of the released rifampicin.

For both types of treatments, cells not exposed to any type of biomaterial were used as control and the proliferation was normalized on day 1 to evaluate the rate of proliferation in time.

**Figure 8:**
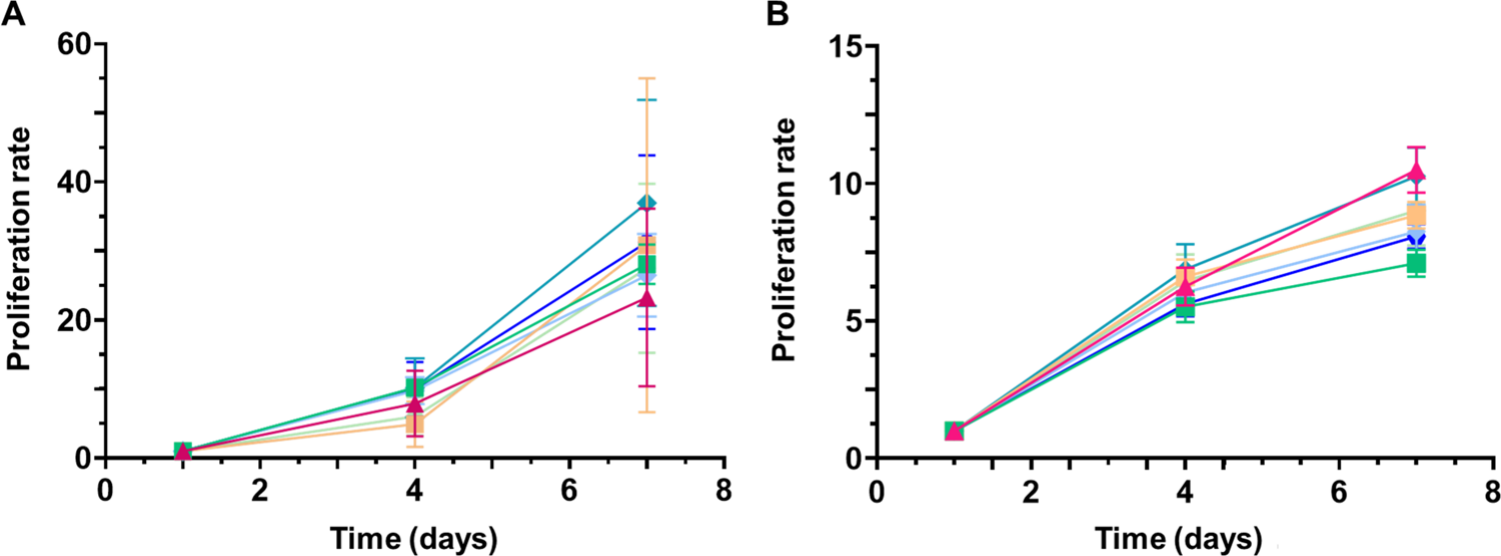
Proliferation rate of NIH/3T3 cells when exposed to not treated (A) and plasma-treated (B) PCL/Rif membranes. In both cases, the same types of samples were examined: CTRL 3T3 (solid magenta triangle up), **CTRL no coax** (solid green box), **CTRL core** (solid light blue diamond), **CTRL shell** (solid blue hexagon), **Rif no coax** (solid orange box), **Rif core** (solid green diamond), **Rif shell** (light green hexagon). The statistical analysis was performed with two-way ANOVA, applying Bonferroni’s correction. No statistically significant differences were detected.

In the case of not treated membranes (Figure 8A), all types of samples revealed a considerable increase in the cell proliferation rate between the fourth and seventh day of culture, with comparable trends. On the other hand, cells in the presence of plasma-treated membranes (Figure 8B) revealed again similar proliferation trends between the different samples examined, but a lower increase between the fourth and seventh day of growth was detected with respect to not treated mats. Such a result was not due to lower biocompatibility of the tested materials, but rather due to cell confluency (as observed by optical microscopy analysis, data not shown), which slowed cell metabolism affecting the Resazurin fluorescence signal. Nevertheless, all of the matrices tested showed good biocompatibility, with no toxic effects deriving from the released rifampicin.

### 3.5 Antibacterial Assays

**Figure 9:**
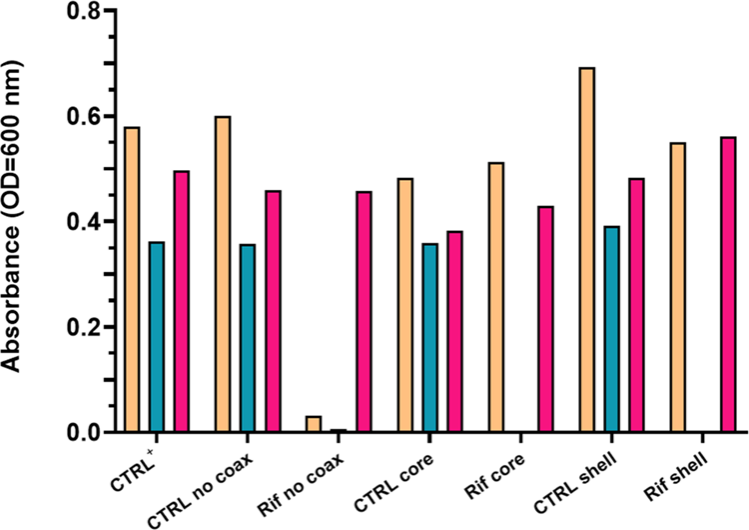
Bacterial growth expressed as absorbance at 600 nm for *E. coli* ATCC 25923 (orange), *S. aureus* ATCC 25922 (light blue), and *P. aeruginosa* ATCC 27853 (pink) cultured in the presence of membranes. Ctrl^+^ indicates bacteria grown without any type of treatment.

The potential inhibitory effect (minimal inhibitory concentration, MIC) of PCL/Rif membranes was preliminarily evaluated toward reference strains of clinically relevant pathogens, namely, *E. coli*, *S. aureus*, and *P. aeruginosa*. Bacterial cultures were grown in a liquid medium in the presence of membranes, whose ability to prevent bacterial proliferation was evaluated by measuring the turbidity of the medium. In particular, the lower the absorbance of the medium at 600 nm, the lower the bacterial growth, and the stronger the antimicrobial effect is. As shown in Figure 9, all of the control PCL electrospun membranes (**CTRL no coax**, **CTRL core**, **CTRL shell**) did not exert any antibacterial effect against all of the tested bacterial strains. In contrast, as regards rifampicin-loaded membranes, all types of samples (**Rif no coax**, **Rif core**, **Rif shell**) strongly inhibited *S. aureus* growth. However, only **Rif no coax** matrices were effective against *E. coli*, while **Rif core** and **Rif shell** were comparable to the control PCL membranes and to the proliferation control (intended as bacteria grown in the absence of any type of treatment), without showing any inhibitory effect. On the other hand, none of the tested samples was able to inhibit *P. aeruginosa* proliferation, with similar results to the non-antibacterial controls, indicating the non-effectiveness of the treatment.

Consequently, with the purpose to evaluate even the possible bactericidal effect of the tested membranes against *S. aureus*, the minimal bactericidal concentration (MBC) assay was performed, withdrawing the bacterial suspension from the wells and spreading it on agar plates. After 18 h of incubation, no bacterial colonies grew, thereby confirming the bactericidal other than the inhibitory effect of **Rif no coax**, **Rif core**, and **Rif shell** mats against *S. aureus* (Figure S4).

## 4 Discussion

Coaxial electrospinning is emerging as a new electrospinning technique, particularly useful in drug inclusion and local delivery of bioactive agents, while actively stimulating tissue regeneration.^75,76^ Indeed, coaxial membranes are characterized by the fibrous structure mimicking the extracellular matrix (ECM) architecture, and their core–shell structure allows the differential incorporation of drugs and bioactive moieties inside the fiber or distribution on its surface, with possible dual drug-release profiles.^77−79^ In this context, the work presented here aims at the exploitation of poly-ε-caprolactone (PCL)-based coaxial fibers as antibacterial drug delivery systems, by alternatively incorporating an antibiotic, namely, rifampicin (Rif), in the core or the shell layer. PCL was chosen for its biocompatibility, bioresorbability, mechanical properties, and versatility, since it is applied for the most varied regenerative purposes, such as bone and cartilage regeneration, wound healing, or nerve regeneration.^80−86^ Due to its hydrophobic behavior, PCL-based membranes need to be modified or treated to increase their hydrophilicity.^87−89^ Among the different techniques, air-plasma treatment revealed to be an effective strategy for activating the PCL mat surface, since it introduces polar groups on the biomaterial surface, increasing its hydrophilicity.^47,90,91^ On the other hand, rifampicin should be particularly useful as a broad-spectrum antibiotic able to penetrate biofilms, being active even against stationary phase bacteria.^92−94^

A comparison between the two types of Rif-loaded coaxial membranes and non-coaxial PCL/Rif nanofibers (named **Rif no coax** or **CTRL no coax**, in the case of the PCL control) was done as a proof of concept, with the purpose of evaluating the effects and benefits of using coaxial fibers over single-needle nanofibers for GTR, where the suppression of the initial drug burst release is a key factor in the success of the biomaterial.^95,96^ The use of single-needle electrospinning requires the exploitation of strategies able to slow the release of the incorporated bioactive compounds.^97^ On the other hand, the use of coaxial membranes provides a simple and effective approach to tuning the release kinetics of the contained drugs.^98^

All types of membranes produced here showed an optimal morphology, with uniform and defect-free fibers. However, the non-coaxial membranes revealed a significantly smaller diameter with respect to coaxial ones. Indeed, whilst a higher applied voltage should decrease the fiber diameter, the greater coaxial fiber dimensions can be ascribed to the employment of an higher concentration PCL solution combined with the same PCL solution used for non-coaxial electrospinning.^99,100^ Afterward, the higher fiber diameter of coaxial membranes clearly influences their surface properties. To analyze them, the wettability of PCL and PCL/Rif membranes was evaluated in the presence of three types of fluids, namely, deionized water (DW), DW added with fetal bovine serum (FBS), and Dulbecco’s modified Eagle’s medium (DMEM). In particular, FBS was added to DW to investigate the possible effects on the wettability of the Rif-loaded membrane, since rifampicin is known for its ability to interact with serum proteins;^101−104^ for the same reason, the DMEM culture medium was tested for its ability to further influence the wettability of the biomaterial in *in vitro* biological environments. As a result, a considerable hydrophobic behavior was observed in all cases, except for **Rif no coax** in the presence of FBS-supplemented water and DMEM; the same behavior was not detectable in the case of **Rif core** and **Rif shell** samples, which were hydrophobic even for these two types of fluids. Such results could be attributed to the morphology of the nanofibers. Indeed, the lower diameter of non-coaxial nanofibers implies a homogeneous rifampicin distribution both in the longitudinal and transverse direction of the fiber. This increases its surface density, resulting in a higher availability for the interaction with serum proteins. Conversely, the distribution of rifampicin on a wider surface, as in the case of **Rif shell** membranes, reduces its surface density and availability,^105−107^ while the incorporation into the core shields any possible interaction, with the hydrophobic shell layer acting as a physical barrier and leading to an overall hydrophobic behavior in the case of coaxial matrices.^108,109^ On the other hand, the activation process flattened the differences between the membranes, leading to a general hydrophilic behavior of all types of samples. This is even confirmed by the assessment of surface free energy, calculated through the Owens–Wendt method, which considers the surface free energy as a function of the polar and dispersive interactions between the solid and the tested liquid. The analysis revealed an increase in the polar component in the case of plasma-treated membranes, thus confirming the higher hydrophilicity of these types of samples with respect to not treated ones.^73,110^

The differential loading of rifampicin into the coaxial and non-coaxial structure does not only influence the surface properties of the material but also the amount of antibiotic released in a more or less short period of time, with consequent differential outcomes on the antibacterial efficacy. The use of coaxial structures should be greatly advantageous in the attempt to tune drug release in time. Therefore, numerous studies have been focused on the use of coaxial fibers for drug loading and release, often including bioactive substances into the core layer, thus allowing a combined effect of protection and prolonged action in time.^111−115^ Other studies focused on the production of coaxial membranes as dual drug delivery systems, to allow a dual-stage release of the same drugs or to modulate and combine the release of different bioactive components.^48,116−118^ In this study, the antibiotic drug was alternatively loaded inside or on the outside of the fiber, with the purpose of evaluating how rifampicin allocation affects its release and antibacterial effects. A further control was given by the rifampicin-loaded non-coaxial structures, which revealed a high release in a short time, with respect to both types of coaxial fibers. Indeed, thanks to the coaxial structure, drug inclusion in the core of fibers protects it from the external environment and slows its release in time, thus allowing a prolonged antibiotic coverage. On the other hand, the incorporation in the shell allowed a faster release of rifampicin with respect to the core, but its distribution on a wider surface was anyway responsible for the reduced availability and slower release than non-coaxial structures.^119,120^ Based on the release kinetics of the drug, a preliminary study of the antibacterial activity was carried out on not treated membranes, testing their ability to inhibit the proliferation of *E. coli*, *S. aureus*, and *P. aeruginosa*, three of the most common bacterial strains accountable for tissue infection and healing failure.^121−127^ As expected, coaxial membranes showed a lower antibacterial activity with respect to non-coaxial structures, being effective only against *S. aureus*, but not toward *E. coli*, which was instead sensitive to **Rif no coax**. This is due to the significantly higher release of rifampicin from non-coaxial matrices, which may be translated into a major contact with bacteria and a stronger effect. On the other hand, the lower amount of available antibiotics in the case of **Rif shell** or its protection inside the core, which determined a slower release, was also responsible for its ineffectiveness against *E. coli*. In fact, rifampicin is well known for its activity against staphylococci, being effective also at low concentrations,^128−130^ but its activity toward Gram-negative bacteria, such as *E. coli*, would require the association with other antibacterial compounds or higher concentrations. For the same reason, none of the analyzed membranes was effective against *P. aeruginosa*.^131−134^ On the other hand, plasma-treated membranes were not tested in this “proof-of-concept study” since the reduced release due to rifampicin degradation upon plasma exposure would have determined a negligible effect toward all of the tested bacterial strains. Given the promising results with *S. aureus*, not only the inhibitory effect but also the bactericidal activity was evaluated in this case, demonstrating the ability of rifampicin to both prevent and counteract bacterial infections.

To prevent any possible toxic effect related to rifampicin released from the functionalized matrix, the biocompatibility of all types of PCL and PCL/Rif membranes was studied by evaluating cell proliferation when exposed to the biomaterial. Both not treated and plasma-treated membranes were tested, revealing in all cases a good proliferation rate. Nevertheless, a lower growth trend was registered in the presence of plasma-treated membranes between the fourth and seventh day of culture, which is not attributed to membrane toxicity but rather to cell confluency. This is supported by the lower amount of rifampicin released from the plasma-treated membranes, which could explain a faster proliferation of cells, reaching confluency earlier compared to the cells in the presence of not treated membranes.

Based on these results, the presented coaxial fiber system emerges as a promising approach for guided tissue regeneration, with an antibacterial potential, which can be tuned by differentially incorporating rifampicin into the core or shell layer of the fiber. The coaxial structure surely allows prolonged release, offering a possible long-term antibiotic coverture, and the inclusion in the core further protects the drug from the external environment avoiding the typical burst release. Moreover, the loading of Rif within the fiber core can be protected from the treatments used to increase the hydrophilicity of the membrane, which can be modulated to preserve the integrity of rifampicin.

Eventually, considering the hydrophilicity and the drug-release properties of the membranes presented here, another medical field of application can be considered, which is the development of wound healing and wound dressing. Indeed, in addition to the importance of the antibacterial properties of these membranes, their hydrophilicity can be exploited for the wound exudate absorption;^135,136^ moreover, the surface charges of the hydrophilic membranes could be used for their functionalization with bioactive compounds^47^ to improve and fasten wound healing.

## 5 Conclusions

Poly-ε-caprolactone (PCL)-based coaxial fibers were synthesized by differentially incorporating an antibiotic drug, namely, rifampicin (Rif), in the core or shell layers, with the aim of tuning its release based on the specific allocation. As a proof concept, coaxial fibers were even compared with Rif-loaded non-coaxial membranes, revealing the effectiveness of the coaxial structure in slowing the release of the antibiotic, which is particularly advantageous to guarantee a prolonged efficacy in time. However, the lower amount of rifampicin available and released in a short time influenced the surface properties of the material, as well as the antibacterial efficacy. On the one hand, rifampicin in the coaxial membranes was not sufficiently available to allow a consistent interaction with serum proteins, with a consequent hydrophobic behavior with respect to non-coaxial mats; on the other hand, the reduced antibiotic release in the first 72 h caused restricted antibacterial action of the coaxial matrices, which were effective only against *S. aureus*. To overcome membrane hydrophobicity, they were subjected to air-plasma treatment, which considerably increased the wettability of the membranes but also caused rifampicin degradation, thereby affecting its release and activity. However, in all cases, membrane biocompatibility was not impaired, both in the presence of not treated or plasma-treated PCL and PCL/Rif matrices, whose lower antibiotic released determined a higher cell proliferation with respect to not treated membranes, thus reaching confluency more quickly. Therefore, the presented coaxial system should be greatly beneficial for numerous regenerative purposes, even if the membrane production and activation process should be optimized to ensure a higher and prolonged antibiotic release as well as efficacy against a wider spectrum of bacteria. The versatility of the process and the opportunity of differentially loading drugs within fibers core or shell offer the opportunity to produce the best performing membrane depending on the specific application, on the tissue damage to be treated, and on the desired drug-release kinetic.

## Supporting Information Available

Contact angle analysis and minimal bactericidal concentration analysis (PDF)

## Abbreviations

**DCM**: dichloromethane
**DMEM**: Dulbecco’s modified Eagle’s medium
**DMF**: *N*,*N*-dimethylformamide
**DW**: deionized water
ECM: extracellular matrix
**FBS**: fetal bovine serum
**GTR**: guided tissue regeneration
**MBC**: minimal bactericidal concentration
**MH**: Mueller–Hinton
**MIC**: minimal inhibitory concentration
**no coax**: non-coaxial
**OD**: optical density
**PBS**: saline phosphate buffer
**PCL**: poly-ε-caprolactone
**Rif**: rifampicin
**γ**_**s**_: total surface free energy
**γ**_**s**_^**d**^: dispersive/hydrophobic component
**γ**_**s**_^**p**^: polar/hydrophilic component
