# Supplementary material for: Tuning the Drug Release from Antibacterial Polycaprolactone/Rifampicin-Based Core–Shell Electrospun Membranes: A Proof of Concept
Source: ACS Appl Mater Interfaces. 2022 Jun 7;14(24):27599–612. doi: 10.1021/acsami.2c04849 (PMC9946292; doi:10.1021/acsami.2c04849)
Supplement: Supplementary file 1 — am2c04849_si_001.pdf [file am2c04849_si_001.pdf]

SUPPORTING INFORMATION

Tuning the Drug Release from Antibacterial Polycaprolactone/Rifampicin-Based Core-Shell Electrospun Membranes - a Proof of Concept

Martina Gruppuso[a], Benedetta Guagnini[a], Luigi Musciacchio[a], Francesca Bellemo[b], Gianluca Turco[a], Davide Porrelli[a1*]

[a] Department of Medicine, Surgery and Health Sciences, University of Trieste, Piazza dell'Ospitale 1, 34129, Trieste, Italy

[b] Department of Engineering and Architecture, University of Trieste, Via Alfonso Valerio, 6/1 - 34127 Trieste, Italy

[1] Present address: Department of Life Sciences, University of Trieste, Via Fleming 31/B, 34127, Trieste, Italy

Email addresses:

Martina Gruppuso: martina.gruppuso@phd.units.it; Benedetta Guagnini: benedetta.guagnini@phd.units.it; Luigi Musiacchio: luigi.musciacchio@phd.units.it; Francesca Bellemo: francesca.bellemo@studenti.units.it; Gianluca Turco: gturco@units.it; Davide Porrelli: dporrelli@units.it

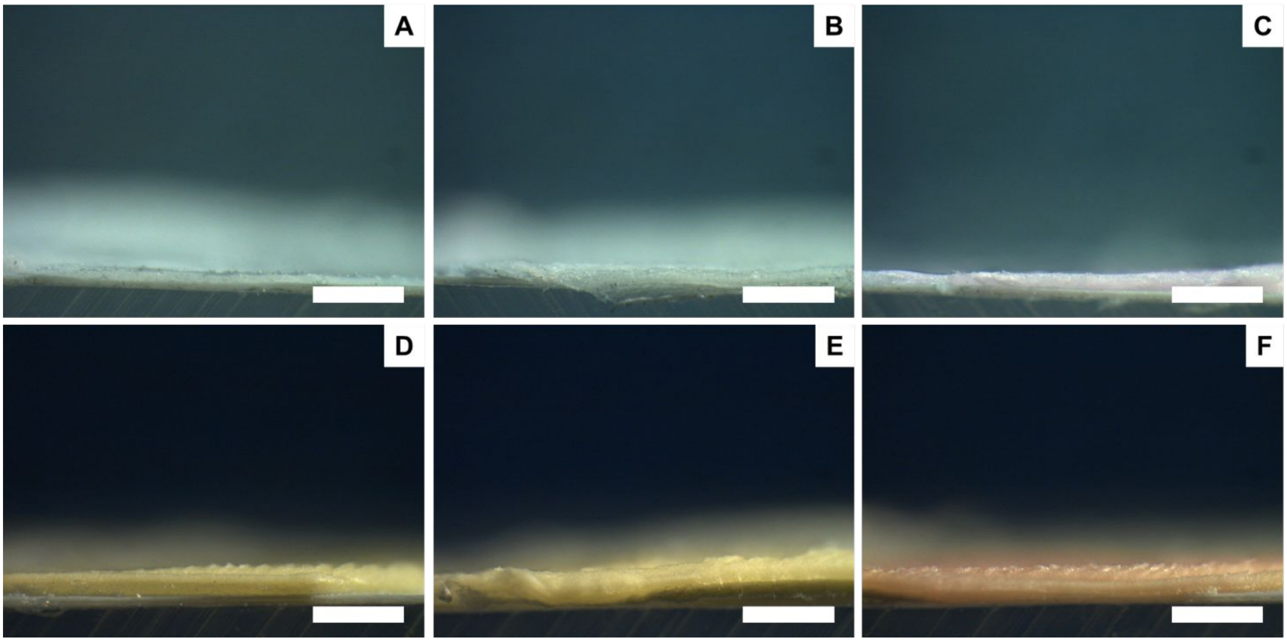

Figure S1. Wettability of plasma-treated **CTRL core** (A-C) and **Rif core** (D-F) membranes evaluated in the presence of three types of fluids: DW (left), DW + 10% FBS (center), DMEM (right). The absence of the drop is due to the immediate fluid absorption upon deposition. Scale bar: 1 mm.

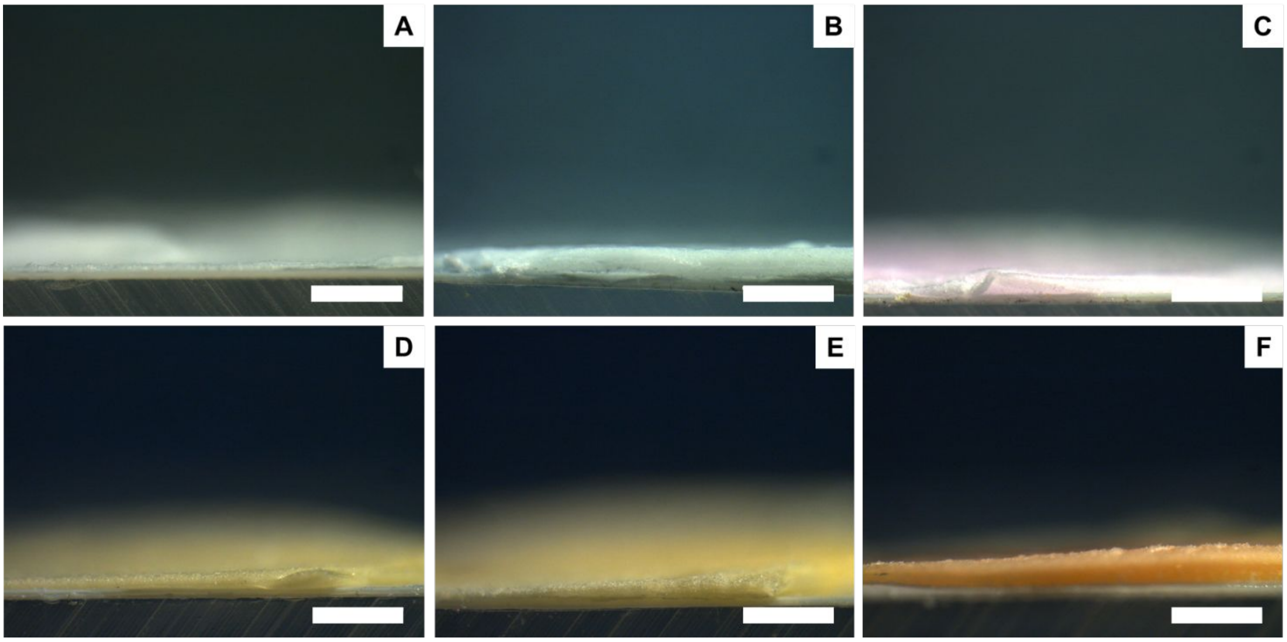

Figure S2. Wettability of plasma-treated **CTRL shell** (A-C) and **Rif shell** (D-F) membranes measured in the presence of three types of fluids: DW (left), DW + 10% FBS (center), DMEM (right). The absence of the drop is due to the immediate fluid absorption upon deposition. Scale bar: 1 mm.

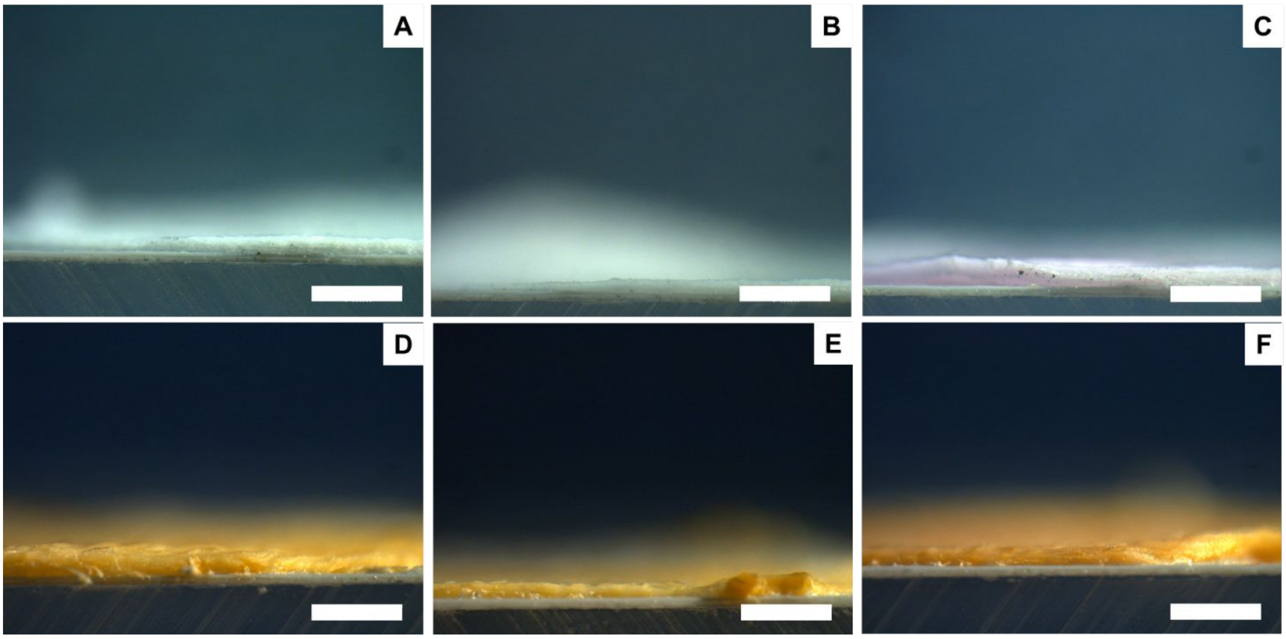

Figure S3. Wettability of plasma-treated **CTRL no coax** (A-C) and **Rif no coax** (D-F) membranes measured in the presence of three types of fluids: DW (left), DW + 10% FBS (center), DMEM (right). The absence of the drop is due to the immediate fluid absorption upon deposition. Scale bar: 1 mm.

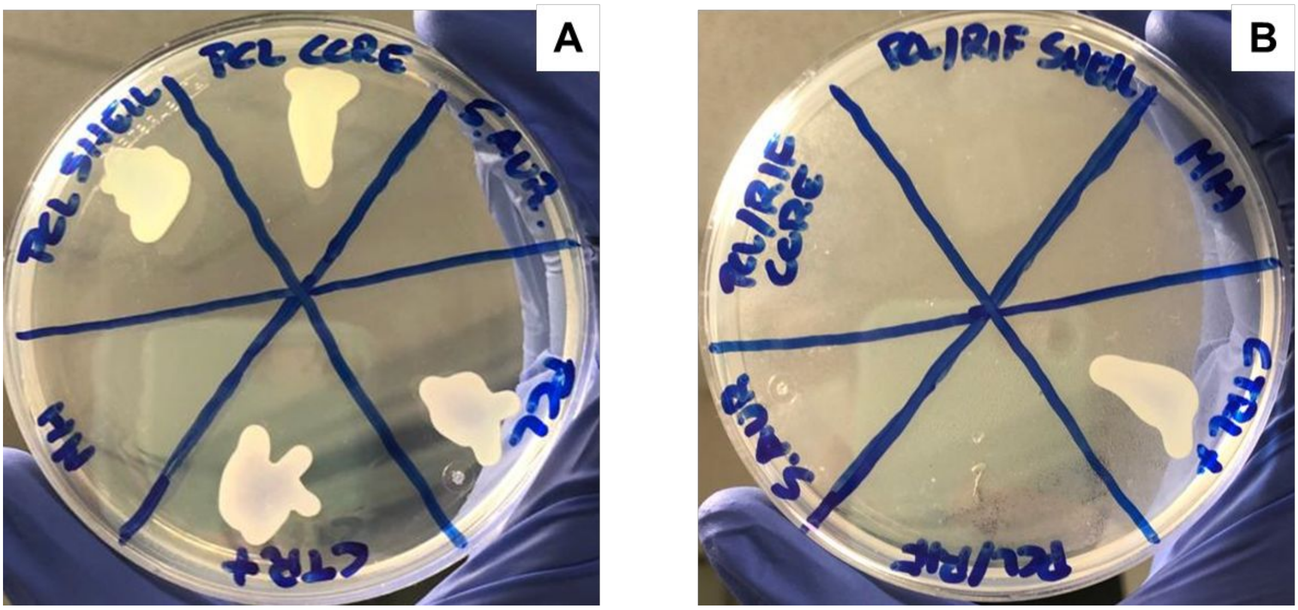

Figure S4. Minimal bactericidal concentration (MBC) assay on PCL membranes (A) and PCL/Rif membranes (B) against *Staphylococcus aureus*. The presence of rifampicin, regardless of its allocation (core, shell, or non-coaxial structure), prevents bacterial colonies growth, which are conversely clearly visible in the case of the growth control ($CTRL^+$) and of the PCL samples without rifampicin.
